# Parents’ Participation in the Sexuality Education of Their Children in Rural Namibia: A Situational Analysis

**DOI:** 10.5539/gjhs.v7n1p35

**Published:** 2014-07-29

**Authors:** Linda Ndeshipandula Lukolo, Agnes van Dyk

**Affiliations:** 1 School of Medicine, Faculty of Health Science, University of Namibia, Windhoek, Namibia; 2 Department of Nursing, Faculty of Health Science, University of Namibia, Windhoek, Namibia

**Keywords:** HIV/AIDS, participation, reproduction, rural Namibia, sexuality, sexuality education, sexual transmitted infection

## Abstract

Talking about sexuality has never been easy in most Namibians cultures and it seems that most parents feel uncomfortable and embarrassed to talk openly with their children about sexuality. They do not participate in the sexuality education of their children, because they believe they are unable to provide quality and adequate sexuality information due to their lack of knowledge about human sexuality or their perceived inability to explain what they do know.

The ultimate purpose of this study was to develop, describe, implement and evaluate an educational programme to empower rural parents to participate in the sexuality education of their children. The study was designed to be qualitative, explorative, descriptive and contextual in nature. It was performed in three phases. Phase 1 consisted of a situational analysis to explore and describe how parents provide sexuality education. Phase 2 consisted of the development of a conceptual framework that facilitated the development of an educational programme. In phase 3 the programme was implemented and evaluated, recommendations were made and conclusions drawn. The main findings revealed two themes: factors influencing parental participation in their children’s sexuality education, and the need for parental participation in their children’s sexuality education.

This article is part of series of three article stems from a study on the topic of sexuality education empowerment programme of rural parents in Namibia. The three articles have the following titles: one: parent’s participation in sexuality education of their children: a situational analysis; two: conceptual framework developments that facilitate the development of an educational programme and three: programme implementation and evaluation. This article dealt with parent’s participation in sexuality education of their children: a situational analysis.

## 1. Introduction and Rationale of the Study

Sexuality education is a lifelong process of acquiring information and forming attitudes, beliefs and values about identity, relationships and intimacy. It encompasses sexuality development, reproductive health, interpersonal relationships, affection, intimacy, body image and gender roles. It also addresses the biological, socio-cultural, psychological and spiritual dimensions of sexuality from the cognitive domain (information), the affective domain (feelings, values and attitudes), and the behavioural domain (communication and decision- making skills) (Taris, 2000).

According to Greathead (2002), parents are the primary sources from which children obtain norms and values. In the context of permissiveness, he argued that parents’ sexual standards are the earliest to which children are exposed and provide the foundation for subsequent sexual development. Schoeberlein (2001) conducted a survey on parent-child communication about sexuality and found that the benefits of such communication may not lie in the actual content of the communication as much as in the attitudes conveyed by parents. He further suggested that if parents influence their children’s attitudes towards sexuality, it is likely that this runs via the transmission of attitudes and values. According to Taris (2005), these findings explain why children who can talk to their parents about sexuality are less likely to engage in sexual activity, and are more responsible in their approaches to sexual activity.

However, Harrison (2000) stated that a large number of children receive considerable misinformation about sexuality issues from their peers and he revealed that parents provide very little direct information about sexuality issues to their children. They also stated that much of the information that children get from their parents is observational and indirect, because children do not get sexuality-related information by having conversations with their parents (Harrison, 2000). According to O’Sullivan (2006) parents do not want to admit that their children are growing up and feel threatened by their children’s sexual development, and thus find it difficult to discuss sexuality-related issues with their children.

In the Namibian context, most parents do not talk openly to their children about issues of sexuality. When the subject of sexuality comes up in these communities, most parents give unclear messages to their children (for girls, “Stay away from boys or you will get pregnant)” without further explanation or discussion on the sexuality matter. Culturally in Namibia, it is a known fact that discussing sexuality issues in society is a very sensitive matter and is considered by many to be a taboo or a sin (Stephenson et al., 2004; Taravera, 2003).

Most Namibian parents do not participate in the sexuality education of their children, because they perceive themselves as being unable to provide quality and adequate sexuality information. Thus many parents, even the best of parents who are committed to the future of their children feel incompetent, inadequate and ill prepared, either factually, emotionally or both, to teach their children about sexuality development, sexuality relations and reproductive health with all its physical, social and ethical implications and consequences (Greathead, 2000; Mtezuka, 1996; Rew, 2005).

Increased incidents of teenage pregnancy in Namibia, and its concomitant adverse effects, are one of the most serious phenomena in a time when more and more children are having children (Teenage parenthood). Teenage pregnancy has a detrimental health, social and economic effect on the teenager, her baby, her parents, and the father of the child, the family and the community at large.

Teenage parenthood is often accompanied by lowered educational attainment and significantly reduced career opportunities and children of young mothers experience more behavioural, cognitive problems, poorer educational achievements and higher teenage pregnancy rate by the time they reach school ages (Jordan et al., 2000).

In light of the rapid spread of HIV, the increase of sexual transmitted infections and teenage pregnancies, lowered educational attainment, psychological and emotional problems, the researcher has become extremely concerned about the future of Namibian children. Therefore the researcher feels that there is an urgent need to promote constructive interpersonal relationships and open communication between parents and their children.

The main purpose of the study was to develop and describe, implement and evaluate an educational programme which will empower rural parents to provide sexuality education for their children. The following objectives were formulated in order to achieve the aim of this study: to explore and describe how rural parents are providing sexuality education for their children, to explore and identify the needs of children regarding sexuality education and to identify and describe challenges encountered by rural parents in the provision of sexuality education of their children,

## 2. Method and Design

The study is a qualitative, descriptive, explorative and contextual in design, and most appropriate to provide information from in-depth descriptions from the data. It was carried out in three phases namely, Phase 1: Situational analysis, phase 2: Conceptual framework and programme development and phase 3, dealt with programme implementation. In this paper, the findings are described and discussed.

This paper dealt with phase one; the situational analysis, involved the qualitative exploration and description on how rural parents are providing sexuality education for their children, and it also explored and described the needs of the children from the sexuality education they detained from their parents and identified challenges experienced by parents in the provision of sexuality education to their children.

During this phase, an explorative, descriptive and contextual investigation by means of focus group interviews, and individual in-depth interviews with rural parents and children were conducted. This helped the researcher to gather information from both parents and children and thus to accommodate the needs of both groups during the development of the programme. The researcher conducted pilot focus group interviews. It was carried out in order to test the practical aspects of a research study and to investigate the feasibility of the proposed study. During the process of conducting the study, the researchers adhered to ethical considerations. Overall permission was obtained from the governors of the regions. Themes were developed based on the findings

### 2.1 Sampling

Purposive sampling was used to select the participants. The population for this study consisted of Oshiwambo speaking rural parents (fathers and mothers) living with a child aged 12-16 years living in the Ohangwena region in Namibia. The participants agreed to participate voluntarily. The sample consisted of seventy (70) participants, of whom fifty (50) were for group discussions and twenty (20) were for individual in-depth interviews. The focus group discussions involved of fifteen mothers (15) and twelve fathers (12), twelve (12) girls and eleven (11) boys. Individual in-depth interviews were conducted twice. Ten (10) parents and ten (10) children were interviewed.

The context for this study is the Ohangwena region. The Ohangwena region is one of the thirteen regions of Namibia and one of the four northern regions. In the north, Ohangwena borders Angola’s Cunene province. Domestically it boarders the following regions: Okavango in the east, Oshikoto in the south, Oshana in the south-west and Omusati in the west. The northern and western parts of the region are the most densely populated. According to the Namibia 2011 population and housing census, Ohangwena had a population of 248,384 growing at an annual rate of 2.4%. The fertility rate was 5.3 children per woman. 1% lived in urban areas while 99% lived in rural areas, and with an area of 10,703 km^2^, the population density was 21.5 persons per km^2^. The census of 2011, found that 15% of the population was under 5 years old, 33% between 5-14 years old, 41% between 15-59 years old, and 9% 60 years and older.

**Figure 1:**
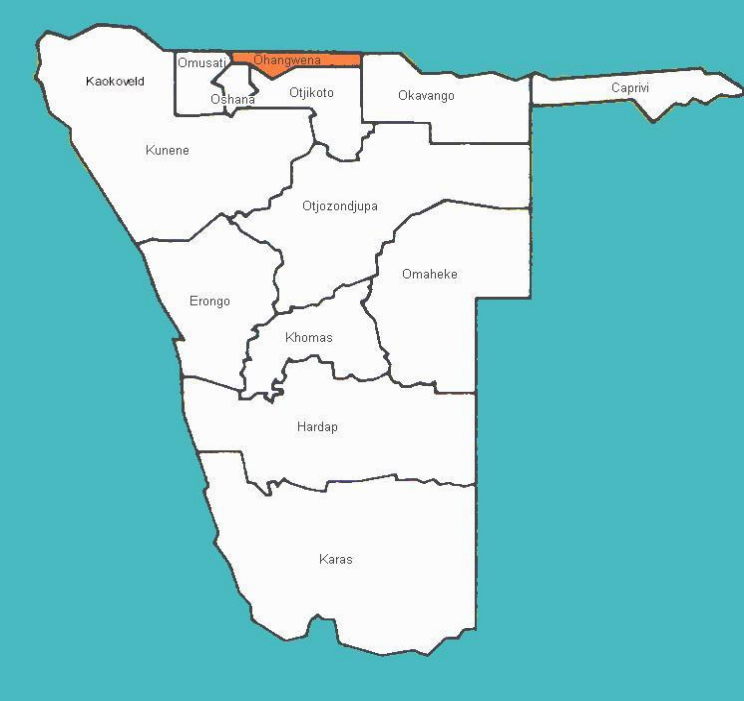
Namibian map

The most commonly spoken language is Oshiwambo, spoken in 97% of households. In terms of education, 53% of girls and 47% of boys between the ages of 6-15 years were attending school, and of those children 15 years and older, 51% had left school, 23% were currently at school, and 23% had never attended school (MOHSS, 2004). The Ohangwena region was selected because rural areas are most less privileged when it comes to the accessibility of health services. Most of the people living in rural areas do not have access to health services, and most of the Namibian youth are residing in northern Namibia. See Figure 1 to locate Ohangwena region.

Cultural norms of this community do not allow parents to talk to their children about sexual related issues, it is considered to be a sin or a taboo. The value attached to culture and the faith that parents put in culture influence their behaviour concerning sexuality-related issues. This influence may have negative effects on health, because it prohibits some positive health practices in the community. In addition, there is fear among some parents that discussion of sexuality issues might encourage children to engage in sexuality activities.

### 2.2 Data Collection

Twenty (20) individual in-depth interviews were conducted with parents and children, as well as, four (4) focus group discussions, consisted of both parents and children, two focus group discussions were for parents and two for children respectively. The reason why selecting individual in-depth and focus group interviews was appropriate, because interviewing is a flexible technique that allows for the exploration of greater depth of meaning (Mouton, 2001; Uys & Basson, 1991). Field notes were also taken to supplement other forms of data collection. A tape recorder was used to store everything that was said for analysis (De Vos, 2007). Data was collected until saturation was reached.

Ohangwena Region

### 2.3 Ethical Considerations

Informed consent was obtained from each participant after the study has been explained to them.

Participation was voluntary and participants retained their right to withdraw at any time without being subjected to any pressure or coercion (Brink, 2007). Consent for tape recorded interviews was obtained. Participants were assured of anonymity and confidentiality, as the researcher was continuously involved with the participants in the field. The researcher undertook to destroy the audiotapes as soon as the data analysis was completed, and this was in fact done.

### 2.4 Trustworthiness

To maintain the true value, applicability, neutrality, validity and reliability in the whole research process, the following were addressed:-

#### Credibility

This was maintained through prolonged engagement, persistent observation, triangulation, and peer group debriefing and member checks.

#### Transferability

through thick descriptive, clear criteria in nominating the sample as well as time sampling, clear description of the participants.

#### Dependability

an external inquiries audit, dense description of the research methods, stepwise replication, triangulation, peer examination and code-record procedure. The researcher gave the transcripts to an external and experts to read through, this was done to ensure the validity and reliability of the study.

#### Confirmability

the researcher safely kept the recorded tape and written documents and notes from the interview for the supervisor to determine whether the conclusion and interpretation can be traced to their sources and if they are supported by the inquiries (Brink, 2007).

## 3. Data Analysis and Findings

Data for this study were analysed by applying Tesch method of qualitative analysis. This is a process of breaking down, examining, comparing and categorising the raw data and at the end putting them together in a new way. The main findings revealed two themes and eight sub-themes. See Table 1.

**Table 1:** Themes and sub-themes

Themes	Sub-themes
Factors influencing parental participation in the sexuality education of their children	▪ Inadequate knowledge on sexuality
	▪ Cultural beliefs of parents
	▪ Negative attitudes and behaviour towards sexuality
	▪ Poor interpersonal relationships between parents and their children
Need for parental participation and involvement in the sexuality education of their children	▪ Need for education and training on issues relating to sexuality and sexuality
	▪ Need for constructive interpersonal relationships between parents and children
	▪ Need to encourage positive behaviour and attitudes towards sexuality
	▪ Need to enhance self-awareness in parents

## 4. Findings and Discussion

These themes and sub-themes were generated and drawn up from the findings. The name chosen were those that seemed most logically related to the data (De Vos et al., 2002; Holliday, 2005; Gorman & Clayton, 2005, Williamson, 2005).

### 4.1 Factors Influencing Parental Participation in Sexuality Education of Their Children

Parental participation in the sexuality education of children is crucial because parents are the primary socialising agents of their children. However, the study found that many parents do not provide adequate sexuality education for their children because they do not know enough about sexuality, they do not know how to explain what they know, and they feel incompetent and embarrassed. The following categories were identified under factors influencing parental participation in the sexuality education of their children.

#### 4.1.1 Inadequate Knowledge on Sexuality

Findings shown that parents found it difficult to provide sexuality education to their children because they do not have proper information, and do not know how or when to start and what to say. Some parents indicated that lack of knowledge about matters of sexuality is one of the obstacles that prevent most of parents from participating in the sexuality education of their children. Parents argued that being uneducated on a certain issue, means that one will not be knowledgeable about the subject matter. The following statements are evidence of that:

“Some of us do not have proper information. Like me, I never attended school. What I can tell my child, the information I have, is only about menstruation. But I do not know how the body works.” (Mother)

The following statements given by children provided further evidence:

I think our parents need information….mh… they need to be educated about these issues, and on how to deal with sexuality-related matters and how to talk to their children. Maybe our parents don’t have proper information because they are not educated or…” (Boy)

These statements are a clear indication that the participants do not have knowledge on sexuality matters. This can be a problem which prevents parents from participating in the sexuality education of their children. Bandura (1992) is of the opinion that initiating conversations about the facts of life may be difficult for some parents because they did not grow up in an environment where the subject was discussed. Some parents may be afraid that they neither know the right answers nor proper amount of information to offer.

However, the results of this study showed schools as the source of information on sexuality. Various authors are of the opinion that, although schools provide information on sexuality in the short term, attitudes do not change to a great extent. Children’s attitudes are more strongly related to values learned at home and to the attitudes of their parents (Blanko, 2000; Bodile, 1994; Shifiona & Mufune, 2000).

The study revealed peers as the second source of information on sexuality. It was the view of children that their peers understand them better than their families do. The closeness of these friendships enable them (children) to share strong and often confusing emotions and experiences, thoughts and dreams, fears and fantasies (Harrison, 2000).

The following statement illustrates this point:

My parents did not tell me anything about sexuality. I only happened to hear from my friends that girls develop breasts and start menstruating when they grow up. We talk freely and better with our friends. But we are children, we do not have information. (Girl)

Bam (1994) states that information on sexuality acquired from peers is likely to be inaccurate, and filled with misconceptions and myths. The sizeable gaps and inconsistencies in children’s knowledge about sexuality are not surprising considering that most of this knowledge comes from peers; and unfortunately peers are often a poor source of sexuality information (Algini & Summers, 2002; Mtezuka, 1996).

#### 4.1.2 Cultural Beliefs of Parents

The results shown that culture promotes inadequate parental participation in the sexuality education of their children because of the the value attached to culture, and, the faith that parents put in culture influence their behaviour concerning sexuality-related issues.

The following quotations are evidence of the above:

According to our traditional beliefs, parents cannot talk to their children about sexuality-related issues, it is considered like a taboo in Namibia. But … nowadays we cannot hide anything because of this rapid spread of HIV-AIDS, and teenage pregnancies with their adverse effects. Parents must talk about what is happening in real life, if they want the HIV/AIDS infection rate to decrease or to stop. They have to be open with their children. (Father)

Another participant responded as follows:

Culture, yes … culture is an obstacle which prevents parents from being open with their children. It is embarrassing to talk about it. Mh mh … you will teach the child to know how to engage in sexuality activity (Father)

The results revealed that some parents did not discuss sexuality issues with their children, because they felt that such discussions would encourage discordant sexuality behavior, and were therefore inappropriate. In addition, fathers stated that they were deterred from discussing sexuality issues because of social-cultural taboos (McCormick & Kosmin, 2003; Resnick et al., 1997). It is a known fact that discussing sexuality issues in society is a very sensitive matter and is considered by many to be a sin or a taboo (Stephenson et al., 2000; Taravera, 2003).

#### 4.1.3 Negative Behaviours and Attitudes Towards Sexuality Education

It is reflected from the findings that negative behaviours and attitudes of parents towards sexuality education are the major sources of inadequate parental participation in the sexuality education of their children.

The following statements serve as evidence:

Me too, I was never told by my parents. And if you heard something and go to ask her for more information, all she will do is chase you away or tell you that you are irritating her. (Girl)

In addition, there is fear among some parents that the discussion of sexuality issues with their children might encourage them to engage in undesirable sexuality activities. This was stated as follows:

In other words, we cannot teach them about sexuality because they will develop ideas which they did not have before. (Father)

Parents were uncomfortable and fearful to raise these issues due to their negative attitude towards sexuality. Many researchers agree that parental attitudes sometimes play an important role in children becoming sexually active. However, the possible effects of parental discussions about sexuality on children’s involvement in sexuality activities appear to be in two opposite forms: discussions can be both an encouragement for children to become sexually active and a discouragement for them to do so (Musarurgwa, 2008).

However, during the discussions most of the participants emphasized that becoming sexually active has various consequences such as sexual transmitted infections, HIV/AIDS, pregnancies and sexuality harassment (MOHSS, 2004).

Sexuality education seeks both to reduce the risks of potentially negative outcomes resulting from sexuality activity, such as unwanted or unplanned pregnancies and sexually transmitted infections, and to enhance the quality of relationships (Ding & Littleton, 2005). It is also about developing young people’s ability to make decisions throughout their entire life. Sexuality education that is effective is sexuality and contributes to the overall aim of sexuality education (MOHSS, 2004; Ritchie, 2006).

#### 4.1.4 Poor interpersonal Relationships Between Parents and Their Children

Poor interpersonal relationships were identified by participants as one of the factors contributing to inadequate parental participation in the sexuality education of their adolescents. The study revealed that poor communication between parents and their children.

The following statement supports this:

Me too, I was never told anything by my parents. When you hear about something pertaining to sexuality and ask your parents for more information, all she will do is chasing you away or tell you that you are irritating her. (Girl) “

Teenagers and parents frequently have different perceptions of the interactions and communication patterns in their family (Rew, 2005). For example, compared to parents, adolescents report less open communication, less closeness between family members, more communication problems, and more power differentials between parents and teenagers (Greathead, 2002; Harrison, 2004).

In general, communication about relationships, feelings, and sensitive issues is the province of mothers (Musarurgwa, 2008, p. 21). Mothers communicate about sexuality more with daughters than with sons. Poor interpersonal relationships lead to poor communication.

### 4.2 Need for Parental Participation and Involvement in the Sexuality Education of Their Children

At home, young people can easily have one-on-one discussions with parents or caregivers; discussions which focus on specific issues, questions or concerns. Sexuality education at home also tends to take place over a long time, and involves many short interactions between parents and children (Taris, 2000).

There are times when young people seem reluctant to talk, but it is important not to interpret any shyness to mean that there is nothing left to talk about. As children get older, parents can take advantage of opportunities provided by things seen on television, which can, for example, be a chance to initiate conversation (Mercer, 2007).

It is also important not to defer dealing with a question or issue for too long as it can suggest unwillingness to talk about it. In order to make the above theme more clear and understandable, it will be discussed under the following categories.

#### 4.2.1 Need for Education and Training of Parents

The study revealed that the need for the education and training of parents on sexuality-related issues was a concern of most of the participants. Children were of the opinion that parents need to be educated about sexuality, and on how to give information to their children because it requires knowledge on sexuality- related issues.

The following comments bear this out:

Our parents need to be educated about this issue of sexuality; perhaps they do not have information about it. (Girl)

Another participant emphasized the need for education when he stated the following:

We do not have knowledge about sexuality matters. I think we need to be educated and directed on how to talk to our children and on what to tell them. (Father)

This can be done by educating parents on how to give sexuality education, and providing them with relevant information on sexuality and sexuality.

*I would like to suggest you place an emphasis on the programme you are going to develop to educate parents to talk to their children, to tell them about what good behaviour is, and what is not acceptable behaviour. (Mother) …Talking to your child does not mean that you actually teach him/her how to do sex, but rather tell him/her how to go about it and all the problems that may result from a certain activities. (Father*) *You need to hold meetings at schools or in churches, and call parents (fathers and mothers) so you can talk to them, and give them information so that they can go and talk to their children about sexuality-related matters. (Boy)*

Participants were of the opinion that sexuality literacy was necessary for parents in order for them to be able to expand their knowledge. According to Taris, (2000) training and development are essential for achieving individual change through learning. He further argues that the focus of learning cannot be confined to knowledge and skills, as individual beliefs, values and attitudes play an important role in facilitating learning and in the successful implementation of planned organisational change (Williams & Wilkins, 2008).

The results of this study revealed the need for parents to be motivated and encouraged to participate in the sexuality education of their children as early as possible. Parental education on sexuality-related issues enables parents to acknowledge the importance of sexuality education and encourage many parents to discuss sexually related issues with their children. Parents should be encourages to talk about sexuality with their children. Participants expressed that fathers should also be motivated to take part in the sexuality education of their children, because they are currently not sufficiently involved in providing sexuality education than mothers. This is the only way children will know how to protect themselves against pregnancies and sexual transmitted infections. Sexuality education through the mass media, often supported by local, regional or national government and non-governmental agencies and departments, can help to raise public awareness of sexuality health issues (Williams & Wilkins, 2008).

In support of the above findings, Harrison, (2002) indicated that children need to have information about the physical and emotional changes associated with puberty, about sexual reproduction, including fertilisation and conception, and about sexually transmitted diseases, including HIV/AIDS.

#### 4.2.2 The need for Constructive Interpersonal Relationships Between Parents and Their Children

Interpersonal relationships can be defined as any of the many and varied relationships which exist within each individual, and between individuals and groups of people and the greater community, and globally. These relationships include, but are not limited to, the individual’s relationship with him/herself, friendships, and romantic, collegial, and community relationships (Harrison, 2002; Rew, 2005).

The results of this study indicate that interpersonal relationships are a key to constructive communication between parents and their children. Participants were of opinion that talking to each other will strengthen and enrich the relationships between parents and their children.

The following were some comments made and suggestions:

Yes, yes… parents should have good relationships with their children. That is the only way that they can discuss sexuality-related matters. Parents must be free and open with their children. (Mother)

I think what is important is good relationships between parents and their children. Parents need to tell their children that every change they notice in their bodies, it is the way children grow. They need to tell them that they are lucky now, because they are being taught how to prevent doing the wrong things. (Father)

In support of the above views of the participants, Goldenberg (2008) also states that sometimes it can be difficult for adults to know when to raise issues, but the important thing is to maintain an open relationship with children which provides them with opportunities to ask questions when they have them. Parents can also be proactive and engage young people in discussions about sexuality and relationships.

The nature of a person’s contribution depends on their relationship, role and expertise in relation to young people. For example, parents are best placed in relation to young people to provide continuity of individual support and education from an early age. The best basis to proceed from is a sound relationship in which young people feel able to ask questions or raise issues if they need to (Hallstead & Reiss, 2003; Steven, 2007; Blanko, 2002).

A study conducted by Fisher (1993) shows that children who reported feeling connected to their parents and family were more likely than other children to delay initiating sexual intercourse. Children who said their families were warm and caring also reported less emotional distress than their peers, they experience less depression and anxiety, and score higher on measures of self-reliance and self-esteem than children whose parents fail to demonstrate these elements (Harrison, 2000; Goldenberg, 2008; Alejandro, Humberto & Agustin, 2008).

Overall, the finding of the importance of general communication styles supports the argument of Kirby, (2007), that if one wishes to improve the sexuality-related communication of parents, one should teach skills pertaining to general communication. This has recently been done with considerable success in an intervention programme designed to improve sexuality-related communication between parents and children (Greathead, 2002).

#### 4.2.3 Need to Encourage Positive Attitudes and Behaviours Towards Sexuality

Most of the participants of this study were of the opinion that positive behaviour and attitudes regarding sexuality-related issues will promote open parent-child communication. Participants believed that they will need to change their behaviour and attitudes towards sexuality-related issues, and should talk to their children about sexuality in order to contribute to the improvement of sexual health amongst young people. This is supported by the following statements:

It is very good and important, because we learn about the good and bad aspects of sexuality activity and about good behaviour. Our behaviour will be determined by our parents’ behaviour. It will prevent us from having unnecessary sexuality activity and teach us how to act responsibly. (Boy)

In support of the above findings, various authors voice the importance of changing negative attitude towards positive attitudes. Maxwell (2006) highlighted that an individual’s commitment to behavioural change is reinforced by being involved in planning, by engaging in self-monitoring, and by conducting frequent “self-awareness checks”. These strengthen commitment, because each check draws attention to a variety of cues making an individual more self-aware of changes in behavior.

He also argued that positive reinforcement can help change behaviour. However, change will not be lasting if an individual does not have an environment that supports activities and the internal motivation to make it a lifelong lifestyle. When these elements are present, reinforcement programmes are useful. Thus motivation is very important to counteract possible resistance. Resistance always hinders change because of the complex nature of change (Maxwell (2006).

Sexuality education is a sensitive subject, young people and sexuality educators can have strong views about the kind of attitudes people should hold and what moral framework should govern people’s behaviours; views which sometimes seem to be at odds (Taris, 2000; Miller, 1998; Musarurgwa, 2008).

#### 4.2.4 Need for Self-Awareness

The participants raised the issue of irresponsible parents, who do not fulfill their important function of bringing up children with socially acceptable behaviours. The following is one of the comments made and suggestions provided:

It seems if our parents do not aware of their responsibility as parents, I think someone should make them aware about this. (Boy). I feel that we parents, needs to know why we are on earth, and to remember that we brought those children on earth, so we must be responsible and take care of our children.(Mother)

Self-awareness enables parents to learn more about themselves and how to deal with daily life situations (Byrne, 2007).O’Sullivan et al (2006) define self-awareness as knowing one’s self and understanding and having insight into one’s own thoughts, emotions and actions. Self-awareness embraces parents sense of knowing who they are, where they come from and the future actions they would like to take in order to reach actualisation, thus allowing them to make sense of their existence.

## 5. Contributions

This study is an original contribution to the body of knowledge on sexuality education. The themes that were developed based on the findings after analysis and interpretation of the results of Phase 1 support the above statement. The study made it possible for parents to receive much needed information pertaining to sexuality education, and created in them an awareness of their responsibilities towards the upbringing of their children, which in turn enabled them to talk to their children about sexuality issues. Parents become aware of themselves and their responsibilities towards the development of their children.

## 6. Conclusion

In this paper, the themes and sub themes identified in phase one; a situational analysis was described. The next paper will be dealing with the development of conceptual framework that facilitates the development of sexuality educational programme to facilitate parental participation in sexuality education of their children.

Parents should fulfill an important function as socializing agents by providing norms and values to their children and that they are primary sources from which children obtain their norms and values. Effective sexuality education also provides young people with an opportunity to explore the reasons why people have sex, and to think about how it involves emotions, respect for oneself and other people and their feelings, decisions and bodies. Young people should have the chance to explore gender differences and how sexuality can influence people’s feelings and options.
